# Bu-Fei-Huo-Xue capsule alleviates bleomycin-induced pulmonary fibrosis in mice through modulating gut microbiota

**DOI:** 10.3389/fphar.2023.1084617

**Published:** 2023-02-08

**Authors:** Haibo Hu, Fengchan Wang, Ping Han, Peng Li, Kun Wang, Huan Song, Guojing Zhao, Yue Li, Xuechao Lu, Weihong Tao, Huantian Cui

**Affiliations:** ^1^ Qingdao Traditional Chinese Medicine Hospital (Qingdao Hiser Hospital), Qingdao University, Qingdao, China; ^2^ Shandong Provincial Key Laboratory of Animal Cell and Developmental Biology, School of Life Sciences, Shandong University, Jinan, Shandong, China

**Keywords:** Bu-Fei-Huo-Xue capsule, pulmonary fibrosis, inflammation, oxidative stress, gut microbiota

## Abstract

**Introduction:** Bu-Fei-Huo-Xue capsule (BFHX) has been used to treat pulmonary fibrosis (PF) in clinic. However, the mechanism of Bu-Fei-Huo-Xue capsule on pulmonary fibrosis remains unclear. Recent studies have shown that the changes in gut microbiota were closely related to the progression of pulmonary fibrosis. Modulating gut microbiota provides new thoughts in the treatment of pulmonary fibrosis.

**Methods:** In this study,a mouse model of pulmonary fibrosis was induced using bleomycin (BLM) and treated with Bu-Fei-Huo-Xue capsule. We firstly evaluated the therapeutic effects of Bu-Fei-Huo-Xue capsule on pulmonary fibrosis model mice. Besides,the anti-inflammatory and anti- oxidative effects of Bu-Fei-Huo-Xue capsule were evaluated. Furthermore, 16S rRNA sequencing was used to observe the changes in gut microbiota in pulmonary fibrosis model mice after Bu-Fei-Huo-Xue capsule treatment.

**Results:** Our results showed that Bu-Fei-Huo-Xue capsule significantly reduced the collagen deposition in pulmonary fibrosis model mice. Bu-Fei-Huo-Xue capsule treatment also reduced the levels and mRNA expression of pro-inflammatory cytokines and inhibited the oxidative stress in lung. 16S rRNA sequencing showed that Bu-Fei-Huo-Xue capsule affected the diversity of gut microbiota and the relative abundances of gut microbiota such as *Lactobacillus*, *Lachnospiraceae*_NK4A136_group, and *Romboutsia*.

**Conclusion:** Our study demonstrated the therapeutic effects of Bu-Fei-Huo-Xue capsule on pulmonary fibrosis. The mechanisms of Bu-Fei-Huo-Xue capsule on pulmonary fibrosis may be associated with regulating gut microbiota.

## Introduction

Pulmonary fibrosis (PF) is the final outcome of numerous interstitial lung diseases, and interstitial fibrosis is the most prominent phenotype in most cases. Most patients with interstitial fibrosis of unknown etiology are eventually diagnosed with chronic hypersensitivity pneumonitis, pulmonary sarcoidosis, or idiopathic interstitial pneumonia (Lederer and Martinez, 2018). In spite of the considerable progress in the field of pharmacological treatment in the last 5 years, the high cost of the drugs makes their practical application unsatisfactory. Therefore, continued search for alternative therapeutic drugs is necessary.

Traditional Chinese medicine (TCM) is reported to have a favorable therapeutic effect on PF (Li and Kan, 2017; Zhang et al., 2021). Mai-Men-Dong decoction can improve lung function in rats with PF by reducing the occurrence of endoplasmic reticulum stress (ERS) and apoptosis in alveolar epithelial type II cells (AECIIs) (Shen et al., 2020). Recent studies have reported that SARS-CoV-2 infection can also cause PF (George et al., 2020). Xuan-Fei-Bai-Du decoction (XFBD) has demonstrated remarkable therapeutic effects on COVID-19, and further studies revealed that XFBD improved bleomycin (BLM)-induced PF and reduced collagen deposition in mice. In addition, XFBD improves PF through the inhibition of the IL-6/STAT3 signaling pathway and the regulation of macrophage polarization (Wang et al., 2021; Wang et al., 2022). Qi-Mai-Fei-Luo-Ping decoction inhibits transforming growth factor (TGF)-β-induced proliferation of A549 cells, attenuates epithelial-mesenchymal transition (EMT), and promotes extracellular matrix (ECM) degradation by inhibiting the TGF-β/Smad3 pathway (Yang et al., 2021). Elucidation of the mechanism of action of TCM in the treatment of PF facilitates the modernization of TCM.

Gut microbiota comprise all intestinal microorganisms and contain diverse populations. Each phylum is distributed in different proportions in different parts of the intestine and they maintain the homeostasis of the gut environment (Jandhyala, 2015). With proposal of the concept of “gut-lung axis,” the study of lung diseases through gut microbiota has become a new research direction (Dang and Marsland, 2019). In recent years, a correlation between gut microbes and PF has been discovered. Compared to healthy subjects, patients with silicosis and progressive PF showed decreased levels of *Actinobacteria*, along with reduced levels of *Devosia, Clostridiales, Alloprevotella,* and *Rikenellaceae_RC9* (Zhou et al., 2019). A study on the correlation between gut microbiota and PF in mice found that *Alloprevotella, Helicobacter, Rikenella,* and *Rikenllaceae RC9* gut group were negatively correlated with the severity of PF, whereas *Dubosiella* and *Parasutterella* were positively correlated with the outcomes of PF (Gong et al., 2021). Regulating gut microbiota may reportedly provide new therapeutic ideas for treating PF. Anthocyanins modulate radiation-induced disturbances in the lung and gut microbiota of mice and reduce radiation-induced lung inflammation and fibrosis (Li and Kan, 2017; Li et al., 2020; Zhang et al., 2021).

Bu-Fei-Huo-Xue capsule (BFHX) consists of *Astragalus mongholicus* Bunge, *Paeonia anomala subsp. veitchii* (Lynch) D.Y.Hong and K.Y.Pan, and *Cullen corylifolium* (L.) Medik. Clinical studies have shown that BFHX can reduce the secretion of inflammatory cytokines in lung tissue and improve the degree of inflammatory damage and fibrosis-like changes in the lung (Jing et al., 2017). However, the underlying mechanism of action remains unclear.

In the present study, a mouse PF model was established using BLM, which was also treated with BFHX. We first confirmed the therapeutic effects of BFHX on PF and chronic inflammation in PF mice, and then detected changes in gut microbiota diversity and abundance in each group of mice using 16s rRNA sequencing.

## Materials and methods

### Reagents

The detailed information of reagents used in this study can be found in Supplementary Materials.

### Preparation and quality control of BFHX

BFHX was obtained from Guangdong Leiyunshang Pharmaceutical Co., Ltd. (Approval No.: Z20030063). Briefly, 30 g of *A. mongholicus* Bunge, 30 g of *P. anomala subsp. veitchii* (Lynch) D.Y.Hong & K.Y.Pan, and 12 g of *C. corylifolium* (L.) Medik were weighed. The herbs were mixed with 576 mL of distilled water. The mixtures were then decocted for 2 h twice and the water extracts of botanical drugs were filtered and evaporated using a rotary vaporization to obtain the drug powder of BFHX. Each capsule contained 0.35 g of drug powder.

High performance liquid chromatography (HPLC) was conducted for the quality control of the drug powder of BFHX. Psoralenoside, isopsoralenoside, psoralen, angelicin and bakuchiol (purchased from Yuanye Bio-Technology, Shanghai) were used as the reference standards for quality control. The experimental conditions of HPLC were shown in the supplementary materials (Supplementary Table S1) and the chromatograms of BFHX and reference standards were shown in Supplementary Figure S1. Three different detection parameters (different chromatographic columns, different mobile phases and different detection wavelengths) within one method were applied to ensure a comprehensive characterization and to countervail the intrinsic limitations of the common fingerprinting methods (Supplementary Tables S2–S5).

### Animals

90 male C57BL/6 mice, weighed 18–20 g, were purchased from Beijing Huafukang Co., Ltd. [Certificate of Approval No.: SCXK (Beijing) 2019-0008]. The feeding environment of animals was shown in Supplementary Materials. The animal study was approved by Animal Medicine and Animal Protection Ethics Committee of Qingdao University (approval no. QDU-AEC-202282).

### Model construction, grouping, and dosing

90 mice were randomly divided into 6 groups as follows: control, model, DXM, low-dose BFHX (LD-BFHX), medium-dose BFHX (MD-BFHX), and high-dose BFHX (HD-BFHX), with 15 mice in each group. For mice in the model, DXM, LD-BFHX, MD-BFHX, and HD-BFHX groups, PF was induced by a single intratracheal injection of 2.5 mg/kg of BLM. Meanwhile, mice in the control group received a single intratracheal injection of an equal volume of PBS. Starting on day 1 after intratracheal injection, the DXM group received 2 mg/kg of DXM *via* intragastric administration on daily basis. The LD-BFHX, MD-BFHX, and HD-BFHX groups received 0.32, 0.63, and 1.26 g/kg of BFHX powder by intragastric administration on daily basis, respectively. Briefly, drug powder of BFHX was weighed and diluted into saline to obtain the BFHX mixture, with the final concentration of 32, 63, and 126 mg/mL respectively. The gavage amount of BFHX mixture was 0.1 mL/10 g. The control and model groups received equal volumes of distilled water *via* intragastric administration on daily basis as vehicle (0.1 mL/10 g), and the entire drug administration process lasted for 21 days. During drug administration, the number of surviving mice in each group was counted daily. The mice were also weighed on days 0, 7, 14, and 21 of administration to observe the changes in body weight. On day 22, after intratracheal injection, all mice were sacrificed and samples were collected.

### Collection of bronchoalveolar lavage fluid (BALF) and lung tissues

After the mice were sacrificed, the thorax was incised and the cervical trachea was fully exposed. After ligation of the left lung, the right lung was lavaged three times with PBS at 4°C, 0.5 ml each time, and the collected fluid was BALF. The lavaged right lung was removed and stored at −80°C. BALF was then centrifuged at 3,000 rpm for 5 min at 4°C, and the supernatant of the first lavage solution was stored at −80°C and analyzed for total protein content using a protein assay kit. Cell pellets from BALF samples were resuspended in PBS for cell counting, and smears were prepared and then underwent to Wright-Giemsa staining to observe various types of inflammatory cells.

### Pathological staining

After BALF collection, the ligated left lung was removed and placed into 4% paraformaldehyde for fixation and embedded in paraffin. The fixed tissue blocks were embedded in paraffin and then cut into 3-μm sections. Hematoxylin and eosin (H&E) staining and Masson’s trichrome staining were routinely performed, and the results were observed under a light microscope. The positive expression of Masson staining was quantified using Image Pro Plus 6.0 software and average optical density (AOD) was calculated.

### Immunohistochemistry

Paraffin sections were made from fixed lung tissue, and endogenous peroxidase was deactivated using methanol-hydrogen peroxide, followed by clearing using PBS and distilled water. Immunohistochemical staining was carried out after antigen retrieval and blocking. The sections were incubated overnight at 4°C with rabbit anti-smooth muscle actin (α-SMA) (1:100) or rabbit anti-transforming growth factor beta 1 (TGF-β1) (1:100). After that, the sections were washed and incubated with secondary antibody (1:10000), washed again. The sections were then mounted after color development and hematoxylin counterstaining. The α-SMA and TGF-β1 protein expression in lung tissue were observed under a light microscope. The expression in the positive regions was quantitatively analyzed using the Image Pro Plus 6.0 software and AOD was calculated.

### Lung tissue biochemical test

We added 100 mg of lung tissue to 900 μL of saline, and homogenized the tissue *via* ultrasonication. The tissue mixture was then centrifuged 3,000 rpm for 15 min at 4°C and the supernatant was collected, where the protein levels in the tissue homogenate were normalized using the BCA kit. Superoxide dismutase (SOD), and glutathione peroxidase (GSH-Px) activities, malondialdehyde (MDA) and hydroxyproline levels were measured in lung tissue using biochemical assay kits. These assays were performed according to the manufacturers’ instructions.

### Enzyme-linked immunosorbent assay (ELISA)

Quantification of interleukin (IL)-1β, IL-6, and tumor necrosis factor alpha (TNF-α) in lung tissue was performed using mouse ELISA kits. The 10% of the lung homogenate supernatant was added to wells coated with the capture antibody. After washing, biotin-labeled detection antibodies were added to each well. After washing, the color development reaction substrate was added and the reaction was terminated after 15 min. The absorbance was read at 450 nm using a microplate reader and the concentration of the corresponding substance to be measured in the sample was calculated after plotting the standard curve.

### Quantitative polymerase chain reaction (qPCR)

RNA extraction kit was used to isolated the total RNA in lung. The purity and concentration of RNA samples were detected by nanodrop. Then the cDNA was synthesized and qPCR was conducted to detect the mRNA expression of *IL-6, IL-1β* and *TNF-α*. *Actb* was used as a loading control. 2^−△△CT^ method was used to calculate the relative expression. The primer sequence was shown in Supplementary Table S6.

### 16S rRNA sequencing

Twenty-one days after BFHX treatment, the fecal contents of mice in control, model and HD-BFHX were collected. The total genomic DNA from the fecal contents of the mice was extracted with the cetyltrimethylammonium bromide (CTAB)/sodium dodecyl sulfate (SDS) method, and the DNA concentration and purity were tested using a 1% agarose gel. DNA was diluted to 1 ng/μL with sterile water based on the concentration. The detailed information about PCR and sequencing data analysis were included in Supplementary Material.

### Statistical analysis

All values were expressed as mean ± standard deviation (SD). Statistical analysis was performed using one-way ANOVA for multiple comparisons. In all analyses, a difference with *p* < 0.05 was considered statistically significant.

## Results

### BFHX improved fibrosis progression in PF mice

**FIGURE 1:**
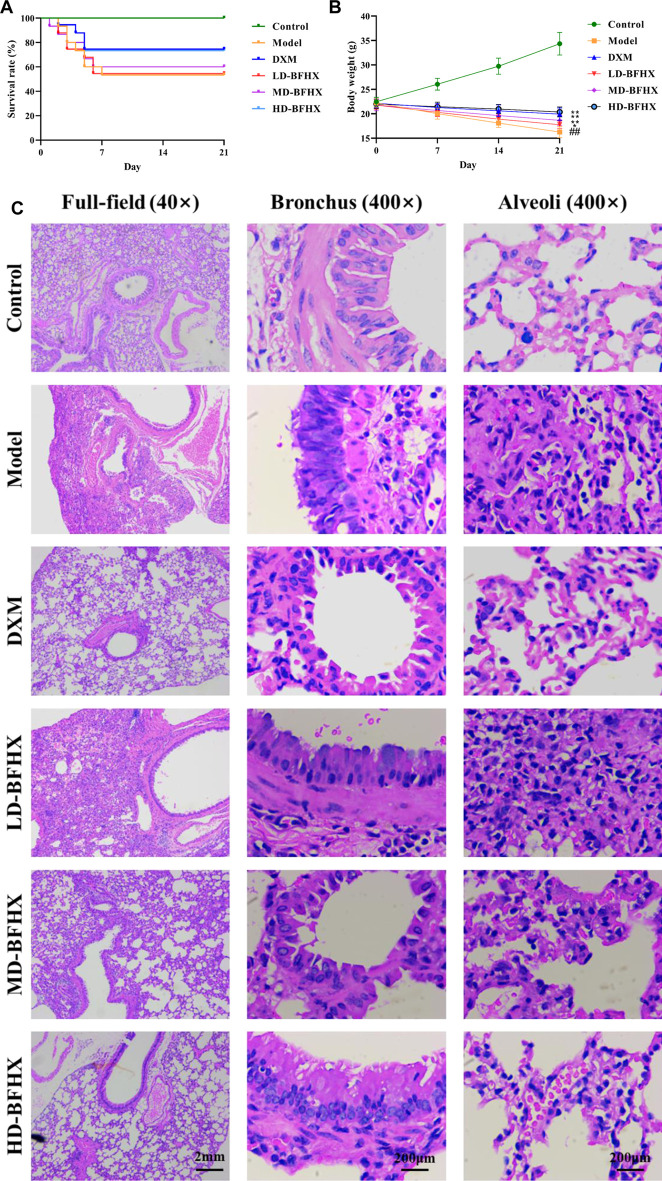
Bu-Fei-Huo-Xue capsule (BFHX) treatment increased the survival rate, ameliorated the body weight loss and reduced the pathological changes in bleomycin (BLM)-induced pulmonary fibrosis (PF) mice. **(A,B)** The survival rate was increased **(A)** and the body weight loss was reduced **(B)** in PF model mice after treated with BFHX; **(C)** H&E staining indicated that BFHX ameliorated the damage of bronchial epithelial cells, the proliferation of fibrous tissue and the infiltration of inflammatory cells in lung (magnification: ×40 for full-field and 400× for bronchus and alveoli) ^##^ *p* < 0.01 compared with the Control group; *p < 0.05 compared with the Model group; **p < 0.01 compared with the Model group Control group (*n* = 15); Model group (*n* = 8); DXM (*n* = 8); LD-BFHX (*n* = 8); MD-BFHX (*n* = 9); HD-BFHX (*n* = 11).

**FIGURE 2:**
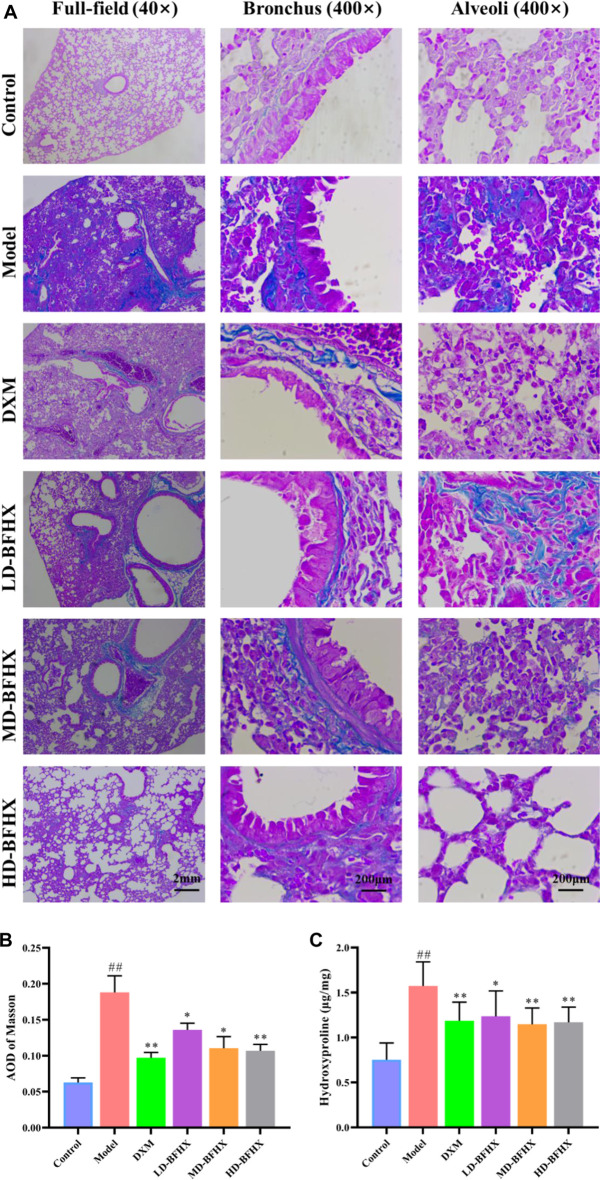
BFHX treatment decreased the deposition of fibrotic contents in lung in PF model mice. **(A,B)** Masson staining showed that BFHX decreased the accumulation of fibrotic contents in lung (magnification: ×40 for full-field and 400× for bronchus and alveoli); **(C)** BFHX reduced the levels of hydroxyproline in lung tissue homogenates.

**FIGURE 3:**
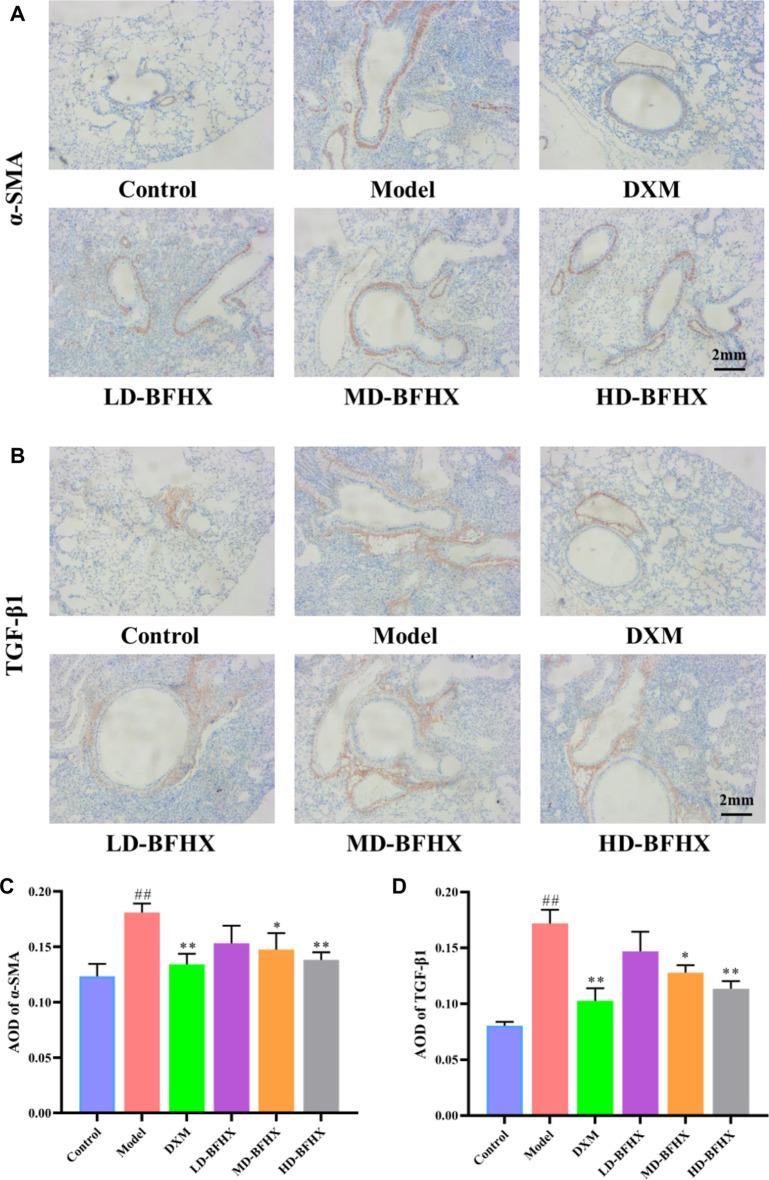
BFHX treatment decreased the expression of α-SMA and TGF-β1 in lung. **(A,C)** Immunohistochemistry showed that BFHX treatment reduced the expression of α-SMA in lung (magnification: ×40). **(B,D)** Immunohistochemistry showed that BFHX treatment reduced the expression of TGF-β1 in lung (magnification: ×40).

After model construction and drug administration, the survival rate was 100% in the control group and 53% in the model group, and DXM and BFHX interventions significantly increased the survival rate of PF mice. The survival rate was 73% in the DXM group, 53% in the LD-BFHX group, 60% in the MD-BFHX group, and 73% in the HD-BFHX group (Figure 1A). Besides, compared with the control group, the body weight of the model group was significantly decreased; compared to the model group, the body weights of mice in the DXM, LD-BFHX, MD-BFHX and HD-BFHX were increased (Figure 1B). H&E staining showed that no alveolar inflammatory exudation and fibrotic lesions were found, and the alveolar structure was clear in the lung tissue of the control group; the structure of bronchial epithelial cells was impaired, the number of interstitial lung cells was increased, fibrous tissue proliferation and fibrosis were present, and alveolar cavity was enlarged and fused in the model group. After the intervention of DXM, LD-BFHX, MD-BFHX and HD-BFHX, lung histopathological changes in mice were reduced, and the lung tissue structure was intact, the alveolar septum was slightly thickened, and inflammatory cell infiltration was reduced (Figure 1C). The results of Masson staining showed that the lung tissues of mice in the control group had normal structural morphology. Compared to the control group, there were significant blue collagen deposits in the interstitium of the model group, which were distributed in large bundles and patches, and the lung tissue fibrosis was severe. Compared to the model group, blue fibrous tissue in the lung tissues of mice was reduced after the intervention of DXM, LD-BFHX, MD-BFHX, and HD-BFHX. The degree of lung tissue fibrosis was significantly improved (Figures 2A, B). The results of hydroxyproline corroborated the degree of pathological changes in lung tissue. Compared to the control group, the model group had significantly higher level of hydroxyproline, and the DXM, LD-BFHX, MD-BFHX, and HD-BFHX groups had lower level of hydroxyproline than the model group (Figure 2C). Besides, immunostaining of lung tissue showed that the expression of α-SMA and TGF-β1 was increased in model group compared with the control, whereas BFHX and DXM treatment reduced the expression of α-SMA and TGF-β1 compared with the model group (Figures 3A–D).

### BFHX ameliorated inflammation and oxidative stress in PF mice

**FIGURE 4:**
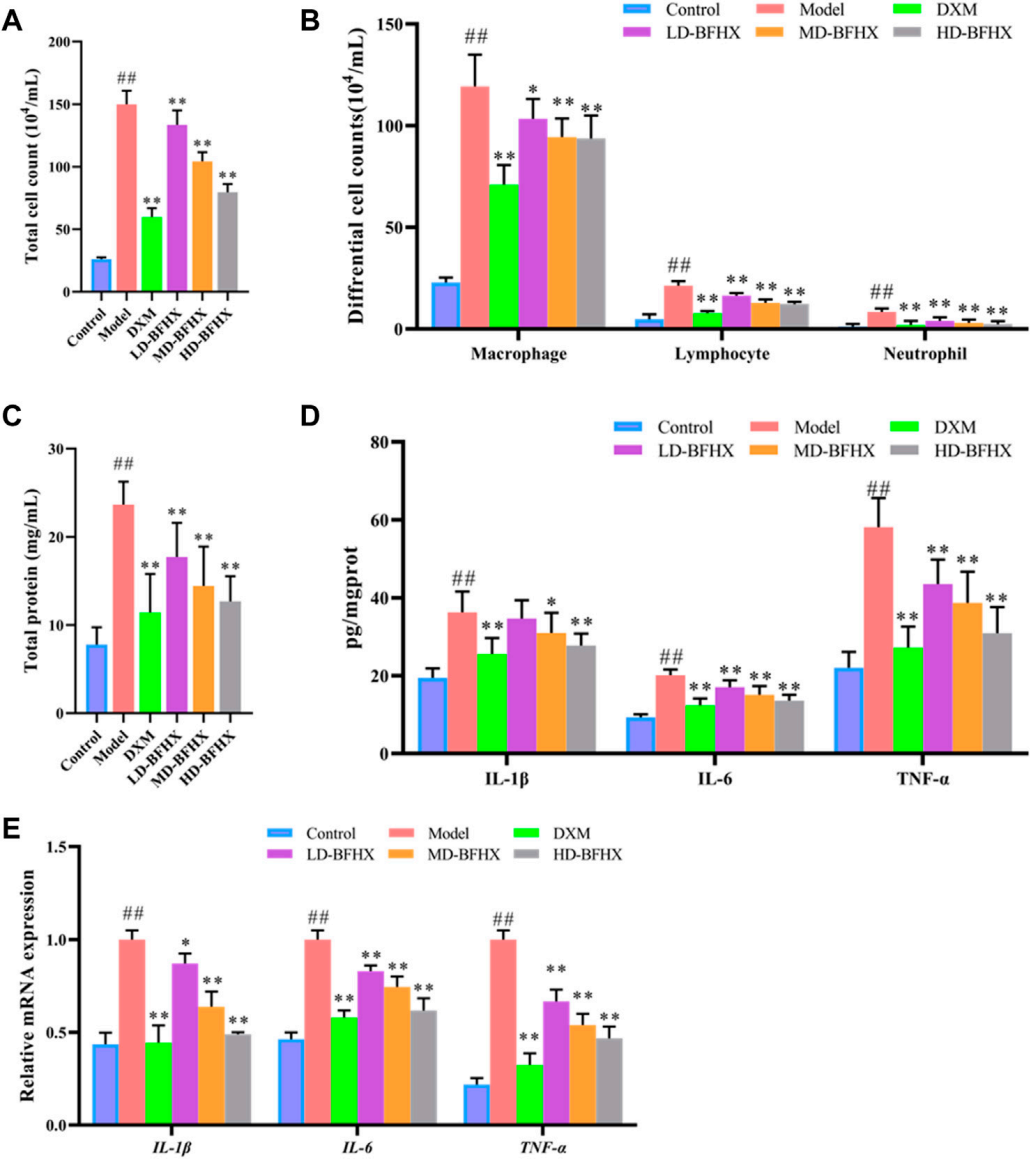
Treatment of BFHX alleviated inflammatory response in PF model mice. **(A–C)** BFHX treatment decreased the total cell counts **(A)**, differential cell counts **(B)**, and total protein concentration **(C)** in bronchoalveolar lavage fluid (BALF) in PF model mice. **(D)** The levels of pro-inflammatory cytokines (IL-1β, IL-6, TNF-α) levels in of lung tissue homogenate were decreased in PF model mice after BFHX treatment. **(E)** qPCR results showed that BFHX treatment downregulated the mRNA expression of *IL-6, IL-1β* and *TNF-α* in lung.

The results of BALF total cell count showed that the total cell count in the model group was significantly greater than that in the control group; the total cell count was reduced in the DXM, LD-BFHX, MD-BFHX, and HD-BFHX groups compared to those in the model group (Figure 4A). Wright-Giemsa staining was used to enumerate macrophages, lymphocytes, and neutrophils in BALF of each group of mice, and the results showed that the counts of macrophages, lymphocytes, and neutrophils were significantly greater in the model group compared to those in the control group. Compared to the model group, the counts of macrophages, lymphocytes, and neutrophils in the DXM, LD-BFHX, MD-BFHX, and HD-BFHX groups and neutrophil counts were reduced (Figure 4B). Total protein quantification showed that total protein concentration in BALF was significantly higher in the model group than in the control group, and DXM, LD-BFHX, MD-BFHX, and HD-BFHX interventions significantly reduced total protein concentration in BALF compared to the model group (Figure 4C). The levels of inflammatory cytokines IL-1β, IL-6, and TNF-α in lung tissue homogenates and BALF were measured using ELISA. The results showed that the levels of inflammatory cytokines, IL-1β, IL-6, and TNF-α, were significantly greater in the lung tissue homogenate in BALF of the model group than in the control group; while IL-1β, IL-6, and TNF-α were decreased in the lung tissue and BALF of DXM, LD-BFHX, MD-BFHX, and HD-BFHX-treated mice, with DXM and HD-BFHX having the most significant reductions (Figure 4D). Moreover, the mRNA expression of *IL-6, IL-1β* and *TNF-α* was upregulated in model group compared with the control group, DXM and BFHX treatment mice showed lower mRNA expression of *IL-6, IL-1β* and *TNF-α* than that in the model group (Figure 4E).

In addition, the effects of BFHX on oxidative stress in PF model mice were evaluated by measuring SOD, GSH-Px activity, and MDA levels in lung tissues of mice in each group. The results showed that the SOD and GSH-Px activities in the lung tissue homogenates of the model group decreased and the MDA level increased compared to the control group; compared to the model group, DXM, MD-BFHX, and HD-BFHX treatment could significantly increase the SOD and GSH-Px activities and decrease the MDA level (Table 1).

**TABLE 1:** Effects of Bu-Fei-Huo-Xue capsule (BFHX) on SOD and GSH-Px activities and MDA levels in lung homogenate.

Group	SOD (U/mg prot)	MDA (nmol/mg prot)	GSH-Px (U/mg prot)
Control	79.93 ± 14.12	1.67 ± 0.25	28.48 ± 7.03
Model	29.88 ± 10.72^##^	3.03 ± 0.39^##^	14.84 ± 3.91^##^
DXM	67.75 ± 13.86**	2.04 ± 0.35**	30.46 ± 4.38**
LD-BFHX	39.50 ± 7.14	2.70 ± 0.49	15.57 ± 5.09
MD-BFHX	60.34 ± 18.01**	2.41 ± 0.30**	19.26 ± 3.67*
HD-BFHX	61.27 ± 8.18**	2.27 ± 0.30**	21.29 ± 4.35**

^##^ *p* < 0.01 compared with the Control group; *p < 0.05 compared with the Model group; **p < 0.01 compared with the model group. Control group (*n* = 15); model group (*n* = 8); DXM (*n* = 8); LD-BFHX (*n* = 8); MD-BFHX (*n* = 9); HD-BFHX (*n* = 11).

In summary, DXM or BFHX treatment improved histopathological changes in the lungs of PF mice, inhibited inflammatory responses, and alleviated oxidative stress. Among the three BFHX groups, the most significant improvement was again seen in the high-dose group. This suggested that BFHX can be a potential therapeutic agent for PF. Therefore, the HD-BFHX group was selected for the subsequent experimental study.

### Results of gut microbiota 16S rRNA sequencing

**FIGURE 5:**
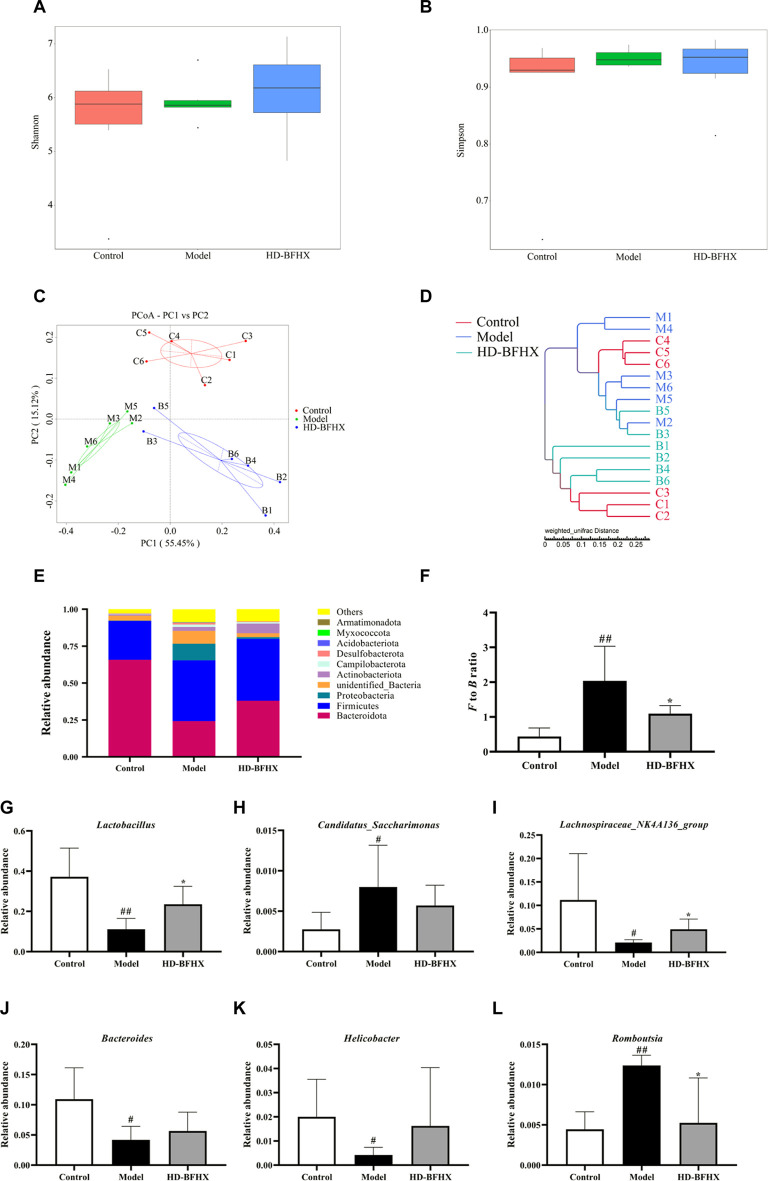
Treatment of high-dose HD-BFHX affected the gut microbiota community in mice with PF. **(A,B)** Shannon **(A)** and Simpson indexes **(B)** were higher in HD-BFHX group than that in the Model group. **(C,D)** Principal Co-ordinates Analysis (PCoA) score plot **(C)** and UPGMA cluster tree **(D)** indicated more similar beta diversity between HD-BFHX **(B)** and Control **(C)** groups than that between the Model (M) and Control groups. **(E**–**L)** At the phylum level, BFHX treatment decreased the *F to B* ratio **(E,F)**; At the genus level, the relative abundances of *Lactobacillus* **(G)** *, Candidatus_Saccharimonas* **(H)** *, Lachnospiraceae_NK4A136_group* **(I)** *, Bacteroides* **(J)** *, Helicobacter* **(K)** *,* and *Romboutsia* **(L)** were changed in PF model mice, BFHX treatment affected the relative abundances of*, Lachnospiraceae_NK4A136_group,* and *Romboutsia* in mice with PF. Control group (*n* = 6); Model group (*n* = 6); HD-BFHX (*n* = 6).

The 16S rRNA sequencing results were used to construct a clustering table with the Amplicon Sequence Variant strategy for subsequent analysis. The *α* diversity of the gut microbial community was assessed by calculating the Shannon and Simpson indices. The results showed that there was no significant difference in Shannon and Simpson indexes in each group (Figures 5A, B). We then calculated the magnitude of differences in the microbial communities between different samples with the Principal Co-ordinates Analysis (PCoA) and UPGMA cluster tree and by assessing their beta diversity. The PCoA and clustering results showed that the sample points of the model group were significantly separated and distant from those of the control group, whereas the sample points of the HD-BFHX group were well separated from those of the model group (Figures 5C, D).

We further compared the relative abundance of gut microbiota to assess microbiota structure. The results showed that *Firmicutes* and *Bacteroidetes* were the dominant taxa in gut microbiota at the phylum level for each group (Figure 5E). The *Firmicutes*/*Bacteroidetes* (*F to B*) ratio was significantly increased in the model group compared to that in the control group, while the *F to B* ratio was significantly decreased after HD-BFHX intervention (Figure 5F). At the genus level, compared to the control group, the relative abundance of *Candidatus_Saccharimonas,* and *Romboutsia* was significantly increased and the relative abundance of *Lactobacillus*, *Lachnospiraceae_NK4A136_group*, *Bacteroides*, and *Helicobacter* was significantly decreased in the model group. Compared to the control group, the relative abundance of *Lactobacillus* and *Lachnospiraceae_NK4A136_group* was significantly increased and the relative abundance of *Romboutsia* was significantly decreased in the HD-BFHX group (Figures 5G–L)

## Discussion

In the present study, we established PF model mice by using BLM. BLM is a chemotherapeutic antibiotic that interrupts the cell cycle, leading to a massive production of free radicals, which causes an inflammatory response and pulmonary toxicity, activation of fibroblasts, and subsequent fibrosis. Therefore, it is widely used in the construction of PF animal models (Moeller et al., 2008; Mouratis and Aidinis, 2011). ECM deposition is the basic pathological feature of PF. Collagen is one of the components of ECM and hydroxyproline is the main component of collagen. Pathological staining of the lungs of mice in the model group revealed extensive fibrosis with structural destruction of the lungs and the appearance of striated fibers, as revealed by H&E and Masson staining. Biochemical assays showed a significant increase in hydroxyproline levels. Epithelial cells in the hypoxic environment created by myofibroblasts undergo apoptosis or EMT, which in turn drives the progression of fibrosis (Yang et al., 2021). Therefore, the expression level of α-SMA can be used to determine the progression of fibrosis (Kyung et al., 2018). Our results found that the expression level of α-SMA was significantly higher in the model group of mice than in the normal group, demonstrating the widespread occurrence of fibrosis. Furthermore, immunohistochemistry revealed the presence of large amounts of TGF-β1 in the lungs of PF mice. DXM is a hormonal drug that is commonly used in the clinical treatment of PF and has been used as a positive control drug in many PF animal studies. Our results suggested that no significant difference existed between the BFHX high-dose group and the active control group intervention in terms of improving lung tissue permeability and pathological changes in PF mice, and these suggested that BFHX has therapeutic effect in PF.

In addition, chronic inflammation is one of the important pathological changes in PF (Thannickal et al., 2004; Du et al., 2019). Under chronic inflammatory conditions, fibroblasts synthesize and release large amounts of ECM, which eventually leads to fibrosis and destruction of normal alveolar structure. Previous studies have reported that macrophages play a key role in PF (Kalluri and Weinberg, 2009; Kishore et al., 2021). During the onset of inflammation, macrophages can rapidly migrate to the site of inflammation, secrete a variety of inflammatory mediators, and promote fibroblast activation and proliferation (Buechler et al., 2021; Du et al., 2022). We measured the counts of total cells, macrophages, lymphocytes, and neutrophils in BALF, and found that cell exudation increased significantly in the model group, and the counts of macrophages, lymphocytes, and neutrophils increased to varying degrees, with macrophages occupying a dominant position. ELISA and qPCR results demonstrated the presence of large amounts of pro-inflammatory cytokines in the lung tissues and BALF of PF model mice than the control group, which is consistent with the results of other studies (Phan and Kunkel, 1992). Our results demonstrated that BFHX reduced the inflammatory response in PF mice.

We also assessed pulmonary oxidative stress by measuring the levels of oxidative stress-related enzyme activities and peroxidation markers. As per our expectations, SOD and GSH-Px activities in the lung tissue of the model group were reduced, whereas MDA levels were increased. This suggested that the lungs were subjected to severe oxidative stress. These sources of stress are associated with several factors besides BLM. Endoplasmic reticulum stress, and peroxisomes are major sources of intracellular reactive oxygen species (ROS); however, macrophages and epithelial cells are the major cellular sources of oxidative stress (Otoupalova et al., 2020). Notably, apoptosis of macrophages releases large amounts of ROS, and this leads to additional macrophage activation and drives PF progression, creating a vicious cycle. Further experiment can be carried out to study the mechanisms of BFHX on PF based on affecting the function of macrophages.

The potential role of gut microbiota in PF has been extensively studied and has been shown to be strongly correlated with inflammation (Bhattacharya et al., 2022). In the present study, the effect of BFHX on the structure and composition of the gut microbiota of PF mice was determined by 16S rRNA sequencing analysis. Alpha diversity of gut microbiota refers to the diversity of microbiota within a specific region or ecosystem and is a comprehensive indicator reflecting the abundance and homogeneity of the microbiota. PF model mice exhibited elevated Shannon and Simpson indices, suggesting elevated alpha diversity in the gut microbiota of PF mice. The beta diversity of mouse gut microbiota was subsequently analyzed using PCoA and cluster analysis, and the overall structure and composition of PF mouse gut microbiota underwent major changes, and BFHX could affect the beta diversity of PF mouse gut microbiota. The results of the analysis of the relative abundance of gut microbiota showed that BFHX could decrease the high *F to B* ratio caused by PF. Changes in the *Firmicutes* to *Bacteroidetes* ratio are intimately associated with many diseases, and *Firmicutes* to *Bacteroidetes* ratio was significantly increased in the PF model. Metabolic disorders and inflammatory responses can be alleviated by decreasing the *F to B* ratio (Trivedi and Barve, 2020; Bhattacharya et al., 2022).

We selected the top few bacteria with the greatest relative total abundance at the genus level for comparison. In PF mice, the relative abundance of *Lactobacillus, Lachnospiraceae_NK4A136_group, Bacteroides,* and *Helicobacter* were significantly decreased, and the relative abundance of *Candidatus_Saccharimonas* and *Romboutsia* were significantly increased. BFHX may have significantly increased the relative abundance of *Lactobacillus, Lachnospiraceae_NK4A136_group* and decreased the relative abundance of *Romboutsia. Lactobacillus,* a natural microorganism with immunomodulatory abilities, has been shown to alleviate respiratory diseases such as asthma in several animal studies and clinical trials (Thannickal et al., 2004; Du et al., 2019). In a previous study, *Lachnospiraceae_NK4A136_group* was significantly and positively correlated with IgE and IL-33 (Wang et al., 2022). However, there was a trend of decreasing abundance of *Lachnospiraceae_NK4A136_group* in the gut microbiota of classical low virulence *Klebsiella pneumoniae* infected mice (Jiang et al., 2022). In a particulate matter-induced lung injury mouse study, the *Lachnospiraceae_NK4A136_group* is likely to be the core gut microorganism playing a protective role (Zhou et al., 2016; Zhao et al., 2021). Our experimental results showed that the relative abundance of *Lachnospiraceae_NK4A136_group* in the gut microbiota of PF mice was significantly reduced, and this was reversed through BFHX intervention. Therefore, the specific role of the *Lachnospiraceae_NK4A136_group* in lung diseases requires further elucidation in the future. *Bacteroides* in gut can metabolize polysaccharides and oligosaccharides to provide nutrients and vitamins to the host and other gut microbiota. However, when *Bacteroides* colonize other sites, they have the potential to become opportunistic pathogens (Zafar and Saier, 2021). *Helicobacter* is a gram-negative spiral bacterium that is closely associated with many gastrointestinal diseases (Graham, 2015). However, controversy exists over the effect of *Helicobacter* on the respiratory system. Some studies have reported a degree of protective effect of *Helicobacter* in the respiratory system; however, other studies have pointed out that there is no negative association between *Helicobacter* and respiratory diseases (Miftahussurur et al., 2017). *Candidatus_Saccharimonas*, a conditional pathogen, was significantly elevated in a colitis-associated carcinogenesis model (Cruzdos et al., 2020). In addition, green tea leaf powder ameliorated high-fat diet-induced abnormalities in lipid metabolism while decreasing its *Candidatus_Saccharimonas* abundance in the gut (Shao et al., 2017). *Romboutsia* was significantly overrepresented in the lung tissue of cancer patients (Ock-Hwa et al., 2022) and positively correlated with the levels of Th2-related factors in the gut microbiota of ovalbumin-induced asthmatic mice, which is consistent with our results and in-depth studies can be conducted using *Romboutsia* as a pathogenic bacterium.

In conclusion, our study demonstrated the therapeutic effects of BFHX on PF. The mechanisms of BFHX on PF may be associated with regulating gut microbiota.

## Data Availability

The datasets presented in this study can be found in online repositories. The names of the repository/repositories and accession number(s) can be found below: NCBI BioProject https://www.ncbi.nlm.nih.gov/bioproject, PRJNA893846.

## Abbreviations

PF, pulmonary fibrosis; TCM, traditional Chinese medicine; ERS, endoplasmic reticulum stress; AECIIs, alveolar epithelial type II cells; XFBD, Xuan-Fei-Bai-Du decoction; BLM, bleomycin; EMT, epithelial-mesenchymal transition; BFHX, Bu-Fei-Huo-Xue capsule; HPLC, high performance liquid chromatography; DXM, dexamethasone; BALF, bronchoalveolar lavage fluid; H&E, hematoxylin and eosin; SOD, superoxide dismutase; GSH-Px, glutathione peroxidase; MDA, malondialdehyde; IL, interleukin; TNF-α, tumor necrosis factor alpha; F to B, Firmicutes/Bacteroidetes.
