# Supplementary material for: Bu-Fei-Huo-Xue capsule alleviates bleomycin-induced pulmonary fibrosis in mice through modulating gut microbiota
Source: Front Pharmacol. 2023 Feb 8;14:1084617. doi: 10.3389/fphar.2023.1084617 (PMC9944029; doi:10.3389/fphar.2023.1084617)
Supplement: Supplementary file 1 [file Table1.DOCX]

**Detailed information of reagents**

Bleomycin (BLM) was obtained from Pfizer Pharmaceuticals Ltd. Dexamethasone (DXM; cat: S17003) was obtained from Shanghai yuanye Biotechnology Co., Ltd. (Shanghai, China). Total Protein (cat: A045-4), superoxide dismutase (SOD; cat: A001-3-2), methane dicarboxylic aldehyde (MDA; cat: A003-1-2), glutathione peroxidase (GSH-Px; cat: A006-2-1) and hydroxyproline (cat: A030-2-1) assay kits were obtained from Nanjing Jiancheng Biological Engineering Institute (Nanjing, China). Enzyme-linked immunosorbent assay (ELISA) kits of mouse interleukin (IL)-1β (cat: EK201B), IL-6 (cat: EK206), TNF-α (cat: EK282) were purchased from Multi Science Biotechnology Co., Ltd. (Hangzhou, China). Rabbit anti- smooth muscle actin (α-SMA, cat: 14395-1-AP) and rabbit anti-transforming growth factor beta 1 (TGF-β1, cat: 21898-1-AP) were purchased from Proteintech (Wuhan, China). RNA extraction kit (cat: DP419), cDNA reverse transcription (cat: R6906), SYBR Green polymerase chain reaction (PCR) amplification (cat: S7516) kits were purchased from Tiangen (Beijing, China).

**Feeding environment of animals**

The mice were housed in specific pathogen-free (SPF)-grade clean environment, at a temperature of 22 ℃ ± 2 ℃, humidity of 50 % ± 15 %, day/night time of 12 h/12 h, and have free access to food and water.

**Detailed 16S rRNA Sequencing**

**Polymerase chain reaction (PCR) amplification and 16S rRNA sequencing**

The primers 338F (5′-ACTCCTACGGGAGGCAGCAG-3′) and 806R (5′-GGACTACHVGGGTWTCTAAT-3′) were used to amplify the V3–V4 regions of the 16S rRNA gene. The PCR amplification system included 10 ng of template DNA, 0.2 µM of forward and reverse primers, and 15 µL Phusion^®^ High-Fidelity PCR Master Mix (New England Biolabs). The reaction conditions were as follows: pre-denaturation at 98°C for 1 minute, denaturation at 95°C for 10 seconds, annealing at 50°C for 30 seconds, and extension at 72°C for 30 seconds, for a total of 15 cycles; with a final holding at 72°C for 5 minutes. The mixture was then stored at 4°C. The mixed PCR products were purified using the Qiagen Gel Extraction Kit (Qiagen, Germany), and tested with 2% agarose gel electrophoresis. The sequencing library was generated using TruSeq® DNA PCR-Free Sample Preparation Kit (Illumina, USA), and the library quality was assessed using Qubit@ 2.0 Fluorometer (Thermo Scientific) and Agilent Bioanalyzer 2100 system. Finally, the library was sequenced on the Illumina NovaSeq platform to obtain 250 bp of paired-end sequences.

**Sequencing data analysis**

The raw sequencing data were assembled and quality-controlled with FLASH (V1.2.7, <http://ccb.jhu.edu/software/FLASH/>) to obtain the final effective tags. The tags were clustered using Uparse (Uparse v7.0.1001, <http://drive5.com/uparse/>) at the 97% similarity level to obtain the operational taxonomic units (OTUs). The OTUs were annotated with taxonomic information against the Mothur algorithm-based Silva database (<http://www.arb-silva.de/>). The MUSCLE software (Version 3.8.31, <http://www.drive5.com/muscle/>) was used for multiple sequence alignment. The OTUs abundance information was normalized according to the sequence number corresponding to the sample with the shortest sequence. Alpha diversity index and beta diversity analysis were subsequently performed. The Wilcoxon rank-sum test was used to test for inter-group differences in the diversity indices; the Kruskal–Wallis rank-sum test (Games–Howell was chosen as the post-hoc test) combined with the multiple testing method FDR were used to screen for differential bacteria, and a difference with *P* < 0.05 indicated statistical significance.

**Quality control of BFHX using high performance liquid chromatography (HPLC)**

0.35 g of contents of BFHX capsule was mixed with 50 mL of methanol followed by extracted with ultrasonic for 30 min and centrifuged at 5000 rpm/min for 15 min. After centrifugation, the supernatant was obtained filtered through a 0.45-μm membrane. Besides, reference standards of psoralenoside, isopsoralenoside, psoralen, angelicin and bakuchiol standards were dissolved in methanol to obtain [reference](javascript:;) [solution](javascript:;). Briefly, all standards were weighed accurately followed by dissolved in methanol. The components of BFHX were analyzed by HPLC (LC-20AD, Shimadzu, Japan). Angilent Eclipse Plus C18 column (3.5μm, 150mm×4.6mm; Agilent Technologies, Inc, American) was used for analysis at 30℃. The mobile phase was 0.1 % phosphoric acid-methanol. The gradient elution procedure is shown in **Table S1**. Column temperature 30 °C; volume flow rate 0.6 mL / min; detection wavelength 310nm (0 ~ 50min), 260nm (50 ~ 80min). The injection volume was 5 μL. (**Figure S1**).

To ensure a comprehensive characterization and to countervail the intrinsic limitations of the common fingerprinting methods, different chromatographic columns, mobile phases and detection wavelengths were used. The relative correction factors (RCF) were calculated for analysis.

The RCF of psoralenoside, isopsoralenoside, angelicin and bakuchiol to psoralen were calculated using psoralen as internal reference substance. The results are shown in Table S2.

Pipette 5 µl of the control solution above and use the Angilent Proshell HPH C18, Angilent Eclipse Plus C18, and Ekzo Nobel Kromasil C18 column for the determination under the chromatographic conditions above. The results are shown in Table S3.

Pipette 5 µl of the control solution above and use 0.05%, 0.1%, 0.2% phosphoric acid/water as mobile phase for the determination under the chromatographic conditions above. The results are shown in Table S4.

Pipette 5 µl of the control solution above for the determination under the chromatographic conditions above. The effects of 308, 310, and 312 nm on the relative correction factors of psoralenoside, isopsoralenoside, and angelicin were investigated respectively. 258, 260, and 262 nm on bakuchiol. The results are shown in Table S5.

The results show that these different detection parameters do not have a significant effect on the RCF.

**Table S1 Gradient elution procedure**

| Time (min) | Methanol (%) | 0.1% phosphoric acid (%) |
| --- | --- | --- |
| 0 | 25 | 75 |
| 10 | 25 | 75 |
| 50 | 50 | 50 |
| 55 | 100 | 0 |
| 65 | 100 | 0 |
| 70 | 25 | 75 |

**Table S2 RCF for each component**

| Volume (μl) | **Relative correction factor** | | | |
| --- | --- | --- | --- | --- |
|  | Psoralenoside | Isopsoralenoside | Angelicin | Bakuchiol |
| 1 | 4.253 | 4.019 | 0.962 | 1.255 |
| 2 | 4.176 | 3.946 | 0.966 | 1.285 |
| 4 | 4.189 | 3.937 | 0.964 | 1.266 |
| 6 | 4.191 | 3.966 | 0.964 | 1.283 |
| 8 | 4.204 | 3.981 | 0.965 | 1.282 |
| 10 | 4.235 | 4.003 | 0.966 | 1.297 |
| Mean | 4.208 | 3.975 | 0.964 | 1.278 |
| RSD | 0.706% | 0.805% | 0.172% | 1.172% |

RSD: relative standard deviation

**Table S3 Effect of different chromatographic columns on RCF**

| Chromatographic column | Relative correction factor | | | |
| --- | --- | --- | --- | --- |
|  | Psoralenoside | Isopsoralenoside | Angelicin | Bakuchiol |
| Proshell HPH C18 | 4.246 | 4.013 | 0.969 | 1.314 |
| Eclipse C18 Plus | 4.208 | 3.975 | 0.965 | 1.278 |
| Kromasil Classic C18 | 4.296 | 4.060 | 0.964 | 1.266 |
| Mean | 4.250 | 4.016 | 0.966 | 1.286 |
| RSD | 1.04% | 1.06% | 0.28% | 1.92% |

RSD: relative standard deviation

**Table S4 Effect of different ratios of phosphoric acid-water on RCF**

| Mobile phase | Relative correction factor | | | |
| --- | --- | --- | --- | --- |
|  | Psoralenoside | Isopsoralenoside | Angelicin | Bakuchiol |
| 0.05% Phosphoric acid/water | 4.436 | 4.096 | 0.961 | 1.296 |
| 0.1% Phosphoric acid/water | 4.563 | 4.261 | 0.964 | 1.293 |
| 0.2% Phosphoric acid/water | 4.654 | 4.398 | 0.965 | 1.288 |
| Mean | 4.551 | 4.252 | 0.963 | 1.292 |
| RSD | 2.406% | 3.555% | 0.227% | 0.288% |

RSD: relative standard deviation

**Table S5 Effect of different detection wavelengths on the RCF**

| Wavelength | Relative correction factor | | | |
| --- | --- | --- | --- | --- |
|  | Psoralenoside | Isopsoralenoside | Angelicin | Bakuchiol |
| 308nm/258nm | 4.272 | 3.856 | 0.974 | 1.317 |
| 310nm/260nm | 4.233 | 4.002 | 0.959 | 1.352 |
| 312nm/262nm | 4.192 | 4.175 | 0.952 | 1.346 |
| Mean | 4.233 | 4.011 | 0.962 | 1.338 |
| RSD | 0.95% | 3.99% | 1.13% | 1.38% |

RSD: relative standard deviation

**Table S6** Primer sequence used for qPCR

| **Genes** | **Primer sequence (5’-3’)** |
| --- | --- |
| *TNF-α* | Forward: GATCGGTCCCCAAAGGGATG |
|  | Reverse: CCACTTGGTGGTTTGTGAGTG |
| *IL-1β* | Forward: AATGCCACCTTTTGACAGTGATG |
|  | Reverse: AGCTTCTCCACAGCCACAAT |
| *IL-6* | Forward: ACAAAGCCAGAGTCCTTCAGAG |
|  | Reverse: TCTGTGACTCCAGCTTATCTCTTG |
| *Actb* | Forward: CCCCTGAACCCTAAGGCCA |
|  | Reverse: ATGGCTACGTACATGGCTGG |

**a**


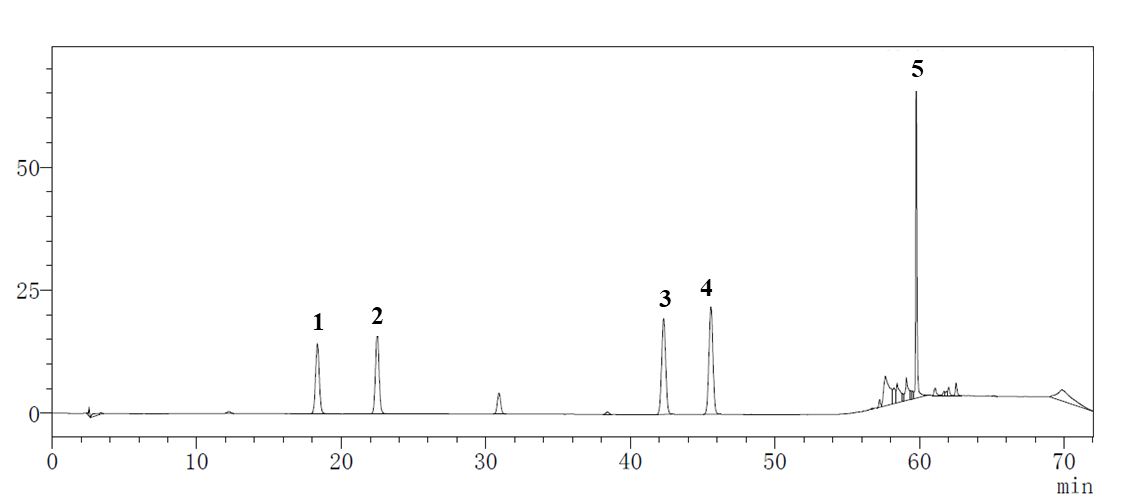


**b**


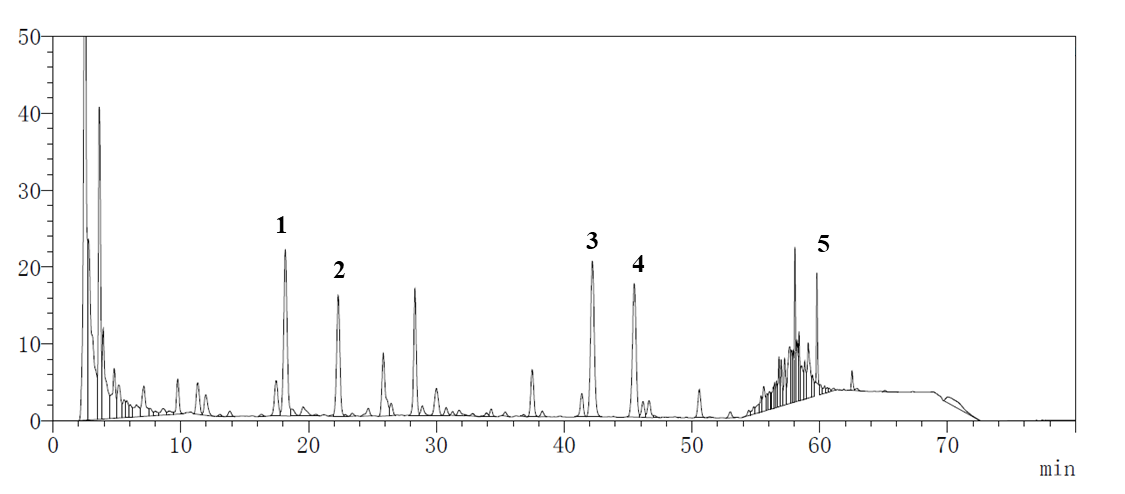


**Figure S1.** The chemical profile of BFHX determined using HPLC analysis. (**a)** The HPLC chromatogram of reference standards. (**b)** The HPLC chromatogram of BFHX. 1 Psoralenoside, 2 Isopsoralenoside, 3 Psoralen, 4 Angelicin, 5 Bakuchiol.
